# Microglial Cannabinoid CB_2_ Receptors in Pain Modulation

**DOI:** 10.3390/ijms24032348

**Published:** 2023-01-25

**Authors:** Kangtai Xu, Yifei Wu, Zhuangzhuang Tian, Yuanfan Xu, Chaoran Wu, Zilong Wang

**Affiliations:** 1 Department of Medical Neuroscience, Key University Laboratory of Metabolism and Health of Guangdong, SUSTech Center for Pain Medicine, School of Medicine, Southern University of Science and Technology, Shenzhen 518055, China; 2 Department of Anesthesiology, Shenzhen People’s Hospital, The First Affiliated Hospital, Southern University of Science and Technology, Shenzhen 518055, China

**Keywords:** the endocannabinoid system, CB_2_ receptors, microglia, pathological pain

## Abstract

Pain, especially chronic pain, can strongly affect patients’ quality of life. Cannabinoids ponhave been reported to produce potent analgesic effects in different preclinical pain models, where they primarily function as agonists of G_i/o_ protein-coupled cannabinoid CB_1_ and CB_2_ receptors. The CB_1_ receptors are abundantly expressed in both the peripheral and central nervous systems. The central activation of CB_1_ receptors is strongly associated with psychotropic adverse effects, thus largely limiting its therapeutic potential. However, the CB_2_ receptors are promising targets for pain treatment without psychotropic adverse effects, as they are primarily expressed in immune cells. Additionally, as the resident immune cells in the central nervous system, microglia are increasingly recognized as critical players in chronic pain. Accumulating evidence has demonstrated that the expression of CB_2_ receptors is significantly increased in activated microglia in the spinal cord, which exerts protective consequences within the surrounding neural circuitry by regulating the activity and function of microglia. In this review, we focused on recent advances in understanding the role of microglial CB_2_ receptors in spinal nociceptive circuitry, highlighting the mechanism of CB_2_ receptors in modulating microglia function and its implications for CB_2_ receptor- selective agonist-mediated analgesia.

## 1. Introduction

Chronic pain is a complex web of emotional experiences and subjective senses that brings patients enormous physical and psychological burdens. Due to the current unmet demand for pain relief, attention has been highly focused on effective analgesics without major central adverse effects. The endocannabinoid system serves as an important neuromodulator signaling, pathway that is deeply involved in the complex modulation of endogenous homeostasis and a variety of pathological processes. Currently, in parallel to the great interest in the endocannabinoid system, cannabinoid compounds, including endocannabinoids, plant-derived, and synthetic cannabinoids, are increasingly recognized as potential therapeutic alternatives for pathological pain, because of their vital roles in modulating nociceptive information processing [1,2].

The biological effects of these cannabinoids are mainly mediated by two endocannabinoid receptors, cannabinoid receptors 1 (CB_1_R) and 2 (CB_2_R). However, currently available cannabis-based medicines are largely targeting CB_1_R, which is detected abundantly in both the peripheral and central nervous systems (PNS and CNS, respectively). Their activation can result in a spectrum of adverse effects, such as the development of tolerance [3], addiction, and psychotomimetic effects [4], thus limiting their therapeutic potential. Subsequently, several strategies have been developed for reducing the central side effects of cannabinoid compounds acting as analgesics, including peripherally restricted CB_1_R agonists, topically applied cannabinoids, cannabinoid metabolic enzyme inhibitors, bifunctional cannabinoids ligands, as well as selective CB_2_R agonists. For example, previously reported VF13, a bifunctional chimeric peptide containing the pharmacophores of the endogenous cannabinoid peptide VD-hemopressin(α) and neuropeptide VF, produced non-tolerance forming antinociception in multiple pain models with reduced cannabinoid-related side effects [5]. In addition, since CB_2_R is primarily expressed in immune cells and relative paucity in neurons, the advantageous profile of CB_2_R agonists devoid of centrally CB_1_R-mediated negative side effects suggests that CB_2_R may represent an attractive target for alleviating pain [6,7]. A large number of CB_2_R selective agonists such as JWH-133, JWH-015, AM1241, and GW405833 have been demonstrated to exert significant antinociception in various preclinical pain model, including inflammatory pain [8,9], postoperative pain [10], neuropathic pain [8,9,11,12,13,14], and bone cancer pain [15,16], without eliciting tolerance [8,17], hypoactivity [18], hypothermia [18,19], catalepsy [19], psychotropic effects and physical dependence [20,21,22]. Therefore, amounts of research efforts focused on the discovery and optimization of synthetic high-affinity CB_2_R agonists to promote selective binding to the CB_2_R are ongoing, such as the acyl hydrazone derivative MDA7, a more promising CB_2_R agonist for severe pain therapy [23,24,25,26].

Historically, CB_2_R was thought to be mainly expressed in peripheral immune cells. However, with the increasing interest in CB_2_R-related research, converging evidence over the last couple of decades has indicated that CB_2_R is also expressed in microglia and some neurons in the CNS. Meanwhile, it is markedly upregulated in activated microglia in response to injury or chronic pain states [10,27,28,29,30,31,32]. Microglia are the macrophage-like cells in the CNS that providing immunomodulatory functions in response to injury or disease. In recent years, increasing studies have demonstrated that spinal microglial activation and microgliosis induced by some inflammatory mediators released from the central nerve terminals of nociceptor neurons play an active role in pathological pain processing [33,34]. Along with rapid morphological changes in microglia, numerous signaling molecules were also altered in microglia in response to microenvironment changes [33,34]. However, strong evidence now supports the hypothesis that the extent of the spinal CB_2_R marked increase in activated microglia may contribute to the shift of microglia to adopt an anti-inflammatory phenotype, with important consequences for the surrounding neural circuitry of nociceptive transmission [35,36,37].

Accumulating studies have shown that targeting spinal CB_2_R represents a promising strategy for pain relief without the classical cannabinoid side effects. However, the underlying mechanisms involved in microglial CB_2_R activation and its contributions to pain processing remain poorly understood. This review will focus on the current research on the roles of microglial CB_2_R in pain processing in vitro and in vivo. We emphasize the involvement of CB_2_R signaling cascades in spinal microglia in the pain processes, as well as the molecular mechanisms involved in mediating the analgesic effects of cannabinoids, especially synthetic CB_2_R agonists.

## 2. The Endocannabinoid System

**Figure 1:**
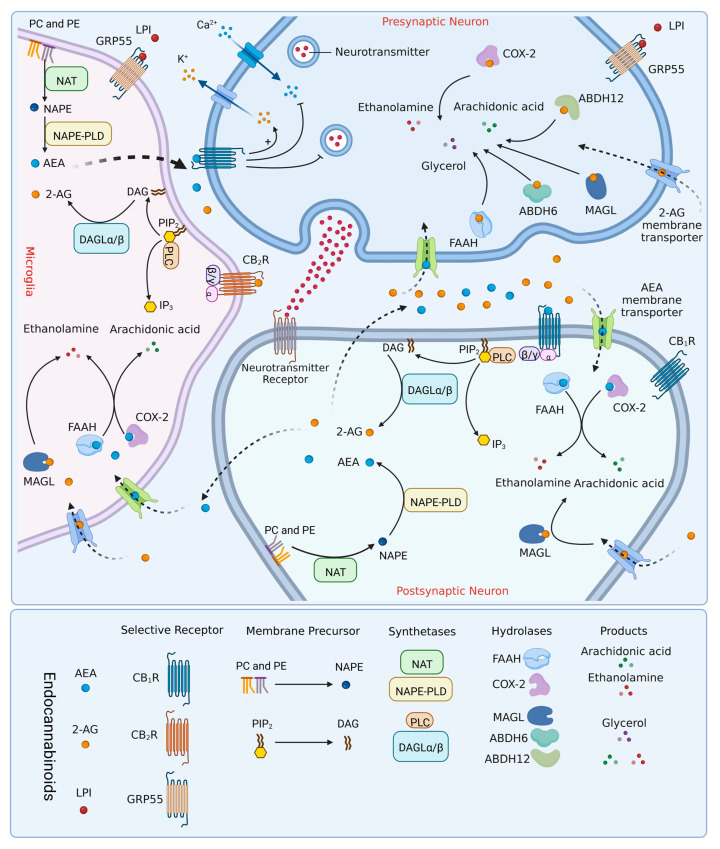
Components of the endocannabinoid system are involved in the main routes of biosynthesis, action, and degradation of endocannabinoids in the nervous system. 2-AG is mainly produced from the hydrolysis of DAG, mediated by two diacylglycerol lipases DAGLα/β. DAG is derived from phosphatidylinositol trisphosphate (PIP_2_), hydrolyzed by PLC. Most AEA appears to be derived from its membrane precursor, NAPE, which is produced by N-acyltransferase (NAT) using phosphatidylethanolamine (PE) and phosphatidylcholine (PC). NAPE can be hydrolyzed by a specific phospholipase D (NAPE-PLD). Microglia may be the primary cellular source of 2-AG and AEA in neuroinflammatory conditions, as they are capable of producing 20 times more endocannabinoids than other glial cells and neurons. AEA and 2-AG benefit from their strong lipid solubility and can be released into the intercellular space through the cell membrane soon after production. AEA mainly plays a role by activating CB_1_R expressed on the presynaptic membrane and postsynaptic membrane. 2-AG can not only activate CB_1_R, but also activate CB_2_R expressed on microglia. After performing their functions, endocannabinoids undergo re-uptake into the neurons and microglia by membrane transporters and are hydrolyzed by different enzymes. 2-AG is degraded by MAGL, ABHD-6, ABHD-12, or COX-2 into arachidonic acid, ethanolamine, and glycerol, while AEA is mainly metabolized by FAAH or COX-2 into arachidonic acid and ethanolamine.

The endocannabinoid system (ECS) is comprised of multiple receptors, endogenous lipid ligands, and various enzymes that regulate endocannabinoid synthesis and degradation, as summarized in Figure 1. As a neuromodulatory system widely involved in lipid signaling responses, the ECS has shown great potential in the development of therapeutic drugs targeting pain management [38]. The endocannabinoids are generated from the hydrolysis of membrane polyunsaturated fatty acids, which is mediated by different enzymes. Arachidonoyl ethanolamide (AEA) and 2-arachidonoyl-glycerol (2-AG) were the first discovered and extensively studied endocannabinoids. Most AEA appears to be derived from its membrane precursor, N-acyl phosphatidyl ethanolamine (NAPE), which is hydrolyzed by N-acyl-phosphatidyl ethanolamine-phospholipase D (NAPE-PLD) [39]. After its release and uptake, the degradation of AEA is primarily via the enzyme fatty acid amino hydrolase (FAAH) [40] or cyclooxygenase-2 (COX-2) [39]. The 2-AG is mainly produced from the hydrolysis of an arachidonoyl-containing phosphatidyl inositol bis-phosphate (PIP_2_) mediated by phosphoinositol-phospholipase C (PLC) and two diacylglycerol lipases α/β (DAGLα/β) [41]. Sequentially, 2-AG is hydrolyzed primarily by monoacylglycerol lipase (MAGL) and to a lesser extent by α/β domain hydrolases (ABDH) 6 and 12. Occasionally, FAAH and COX-2 also degrade 2-AG under some conditions [42,43,44]. In addition to the best-known 2-AG and AEA, some neuropeptides are also considered to be endocannabinoids and have been described to have CB_1_R and CB_2_R activity, such as hemopressin, a fragment derived from the α-chain of hemoglobin [45,46,47,48].

The CB_1_R and CB_2_R are the two identified and best-characterized cannabinoid receptor subtypes. Both are seven transmembrane inhibitory G_i/o_ protein-coupled receptors involved in classical signal transduction pathways, including inhibition of adenylyl cyclase (AC) activity, reduction of cyclic adenosine monophosphate (cAMP) production, as well as signal pathways associated with mitogen-activated protein kinases (MAPK) [49]. The 2-AG acts as a high efficacy full agonist for both CB_1_R and CB_2_R. However, AEA exhibits low efficacy and can act as a partial agonist preferentially for CB_1_R with 4-fold selectivity compared with CB_2_R [50,51,52]. In addition, previous studies demonstrated that the putative non-CB_1_R/CB_2_R orphan receptor GPR55 might represent the third cannabinoid receptor (CB_3_R), though GPR55 shares a low amino acid sequence with both CB_1_R (13.5%) and CB_2_R (14.4%) [53,54]. Subsequently, lysophosphatidylinositol (LPI) is identified as an endogenous agonist of GPR55 [55], while the physiological role and pharmacology of GPR55 remain unclear [56]. Recent studies in animals suggest that GPR55 also plays an important regulatory role in pain signal processing in the CNS [57].

It is acknowledged that CB_1_R is abundantly expressed in neurons of the brain cortex, cerebellum, basal ganglia, and hippocampus within CNS areas. These brain areas not only regulate movement and memory functions, but also intricate pain signaling, which accounts for the impact of CB_1_R agonists on psychoactivity, cognition, memory, and locomotive activity [57,58,59]. In the spinal cord, CB_1_R was found in the glutamatergic interneurons, glycinergic interneurons, GABAergic inhibitory interneurons, and a subset of spinal projection neurons [60,61]. The CB_1_R functions primarily by modulating neuropeptide and neurotransmitter release to inhibit synaptic transmission in the brain and spinal cord. Activation of CB_1_R results in the activation of inwardly rectifying K^+^ channels, promoting cell hyperpolarization, which leads to the inhibition of the presynaptic neurons. Meanwhile, activation of CB_1_R can inhibit Ca^2+^ channels to decrease neurotransmitter release [62] (Figure 1). Additionally, CB_1_R has been detected in the peripheral nervous system like the dorsal root ganglion (DRG) and other tissues, such as the spleen [63], leukocytes [64], tonsils [65], skin [66], testes, heart, lungs, small intestine, urinary bladder, prostate, uterus, ovary, and vas deferens [67,68,69]. Recently, CB_1_R has also been reported as functionally expressed in different glial cells, specifically at a low level in microglia and mainly in different neuronal subtypes. Whether and how CB_1_R regulates microglial function remains unclear. Moreover, CB_1_R is also expressed in astrocytes, shaping synaptic transmission and memory [35,70,71,72,73].

The CB_2_R shows a distinctly different distribution compared to the CB_1_R, which was initially identified predominantly at the periphery, mainly in immunocytes, such as lymphocytes, neutrophils, leukocytes CD8 and CD4, and macrophages [2,4,35,74,75,76,77] in various tissues, such as the liver [78], pancreas [79], bone [80], skin [66], spleen, and vascular elements [81]. Subsequent data indicated that CB_2_R can also be detected in nervous systems, but the expression level of CB_2_R is 100-fold lower than that of CB_1_R under normal physiological conditions, which also suggests that the activation of CB_2_R may have a different pharmacological profile and not be involved in the unwanted cannabis-like effects [82]. Within the CNS, the CB_2_R appears to be expressed by some neurons specifically within the cortex, striatum, hippocampus, amygdala, olfactory, cerebellum, thalamus, spinal nuclei, and substantia nigra [69,83,84]. Meanwhile, the accumulating reports showed that CB_2_R can also be found in brain glial cells, particularly microglia [85,86,87]. When compared with CB_1_R, CB_2_R exhibit one unique feature that is a dynamic expression manner under certain pathological conditions (e.g., inflammation, anxiety, epilepsy, and nerve injury) in neurons and microglia, suggesting the alteration of CB_2_R expression and function are closely related to these neurological diseases [9,32,83,84,88,89,90,91].

Notably, the spinal cord distribution of the CB_2_R (mRNA or protein) has been identified primarily in microglia, especially activated microglia, and poorly expressed in neurons by the preponderance of evidence using quantitative real-time PCR [6,9], in situ hybridization [28], western blotting [92,93], immunohistochemical staining [17], or single-cell sequencing [60]. Intriguingly, similar to peripheral CB_2_R, which is mainly detected in immune cells, central CB_2_R is predominantly expressed in microglia, which indicates that CB_2_R is critically involved in the modulation of immune-related responses, including immune-neuron interactions and the occurrence of neuroinflammatory and pathological pain [94].

## 3. Microglia Express the CB_2_R-Related Functional Endocannabinoid System

In acting as the central custodians protected by the blood-brain barrier, microglia are derived from primitive macrophages that emanate from the embryonic yolk sac and invade the CNS via the circulatory system during development [95,96]. Over recent decades, a large amount of strong evidence has supported the hypothesis that microglia-dependent phagocytosis/degradation and neuroimmune response critically contributes to the alterations in synaptic remodeling and signaling pathways for chronic pain development [33,34,97,98]. In order to maintain stability in an ever-changing environment, microglia adopt different phenotype states characterized by distinct morphological types to perform their functions.

In the healthy adult CNS, homeostatic microglia with a small soma and highly ramified processes classically display a “resting” phenotype to continuously palpate the environment [95]. In nerve injury or chronic pain conditions, spinal microglia may rapidly proliferate and become activated to a pro-inflammatory (M1, classically activated state) phenotype with shorter processes and hypertrophic soma to respond to some inflammatory mediators, including neurotransmitters (e.g., calcitonin gene-related peptide, CGRP), growth factors (e.g., colony-stimulating factor-1, CSF-1), adenosine triphosphate (ATP), chemokines (e.g., CX3CL1), and enzymes (e.g., Caspase-6), released from central nerve terminals of nociceptor neurons [99,100,101]. Meanwhile, microglia produce and secrete various pro-inflammatory mediators, including interleukin 1β (IL-1β), IL-6, interferon γ (IFN-γ), tumor necrosis factor α (TNF-α), and prostaglandin E_2_ (PGE_2_), which can promote immunological actions and can also act on neurons to lead to central sensitization of surrounding neuronal circuits, contributing to the manifestation of chronic pain [102]. 

In addition, after this M1 response, aimed at eliminating noxious stimuli and restraining the initial inflammation from the area, the resolution of the inflammatory process is essential to bypass neurotoxicity and chronic inflammation [103]. For this resolving phase to occur, a shift in microglia phenotype along a spectrum of activation states from pro-inflammatory to phagocytic anti-inflammatory (M2, alternative activation state) is typically observed, which could increase the production of anti-inflammatory mediators, including transforming growth factor (TGF-β), interleukin 10 (IL-10), IL-13, and IL-4 [104,105]. This process can dampen the pro-inflammatory cytokine levels and promote the resolution of inflammation and the recovery of tissue homeostasis [105,106].

Previous studies in CB_2_R^−/−^ mice have demonstrated that an exacerbated microgliosis occurs in the spinal cord after peripheral nerve injury [29,107], which indicates the relevant role of CB_2_R in controlling microglial proliferation and reactivity. Indeed, accumulating reports have shown that microglia express functional endocannabinoid signaling, which could modulate the activity of microglia to restore CNS homeostasis during pathological neuroinflammatory conditions [108,109]. Firstly, classic studies have shown that cultured microglia from humans [103,110], rats [77,111], or mouse tissues [9,85] and the BV-2 microglia cell line [85], express large amounts of CB_2_R. As previously mentioned in Section 2, CB_2_R is predominantly expressed in activated microglia within the CNS, which is consistent with CB_2_R upregulation in activated macrophages in the peripheral [77,112]. By contrast, the expression of CB_1_R in microglia is ambiguous. Preliminary evidence from early studies suggested that CB_1_R is expressed in culture microglia prepared from mollusks and rodents [111,113], but not in humans [110]. However, it has recently been shown that, unlike *Cnr2* (CB_2_R) transcripts, *Cnr1* (CB_1_R) expression was not detected in naïve microglia by microfluidic RT-qPCR [114]. These results require further functional and anatomical evidence to investigate whether CB_1_R can regulate microglial function. To date, Agnès Nadjar et al. have provided the only evidence of CB_1_R functional expression on microglia [115].Their results showed that neither male nor female animals exhibited any peculiar behavioral phenotype when CB_1_R was selectively deleted in CX3CR1-positive cells, including motor activity, anxiety levels, learning, and memory. These results suggested that there is a possibility that low expression of CB_1_R in microglia has no clear role on microglia function. Futhermore, Maciej Pietr et al. found that GPR55 is significantly expressed in both the BV-2 cells and primary mouse microglia. Their expression pattern response to the state of microglia is very similar to that of CB_2_R, suggesting it might also be involved in the neuroinflammatory process [116].

Microglia not only express CB_2_R responding to cannabinoid ligands but also produce and inactivate the endocannabinoids AEA and 2-AG by the complex cellular machinery [111,117,118]. The endocannabinoids are highly lipophilic molecules, that are naturally synthesized from glia and neuron membrane phospholipids during physiological events in the CNS. As a result, endocannabinoids are released immediately after their production to stimulate CB_1_R and CB_2_R, and in turn, they are inactivated by re-uptake and the ensuing hydrolysis [119,120]. Notably, under neuroinflammatory conditions in vitro, microglia are capable of producing 20 times more endocannabinoids than other glial cells and neurons. Therefore, microglia may be the primary cellular source of endocannabinoids in vivo [35,85]. Note that the expression of AEA and 2-AG is significantly upregulated when microglia are activated and switch to a protective phenotype (M2) under pathological conditions [68,103,121]. Further studies have demonstrated that the production of 2-AG and AEA is dependent on sustained rises in intracellular Ca^2+^ concentration, which may be mediated by the activation of ionotropic P_2_X purinergic receptors P_2_X_4_ and P_2_X_7_ [118,122]. The molecular mechanism underlying this process involves the fact that an increase in intracellular Ca^2+^ can directly increase NAPE-PLD and DAGL activity while inhibiting MAGL activity in microglia [85,123,124]. The inverse sensitivity of DAGL and MAGL to Ca^2+^ constitutes a primitive and efficient modality for microglia to continuously accumulate 2-AG [118,125]. Additionally, microglia also express the metabolic enzyme FAAH, which is responsible for the degradation of 2-AG and AEA [123]. Several reports have detailed that under neuropathological conditions, activated spinal microglia may produce significantly more 2-AG and AEA by upregulating DAGL and NAPE-PLD, while downregulating FAAH [103,126,127]. Meanwhile, it has been suggested that the exposure of human or rat microglia to 2-AG and AEA at low concentrations increases the expression of the M2 microglial marker arginase 1 (Arg-1), together with other markers such as suppressor of cytokine signaling 3 (SOCS_3_) [103]. These results support that the production and actions of endocannabinoids are closely related to the phenotype of microglia, and the endocannabinoid system also plays an important role in microglial immunomodulation by inducing an M2 phenotype. It also explains that some of the neuroprotective effects of the endocannabinoid system in diverse pathological states of inflammatory models may be mediated by their immunomodulatory actions, such as disrupting pro-inflammatory processes [128,129,130].

The increased production of endocannabinoids may also upregulate the expression of microglial CB_2_R, which in turn may activate more CB_2_R to dampen nociceptive signaling cascades, amplifying the anti-inflammatory responses [103]. Notably, the activation of CB_2_R has been reported to transform microglia from the M1 to the M2 phenotype [131,132], suggesting that CB_2_R signaling is very important for microglia to polarize towards the M2 phenotype with phagocytic capacity by morphology alterations [103]. These findings are consistent with previous studies that showed that upregulation of CB_2_R in activated microglia has been associated with improvement of the disease consequences in specific neuroinflammatory conditions [126,133,134].

**Figure 2:**
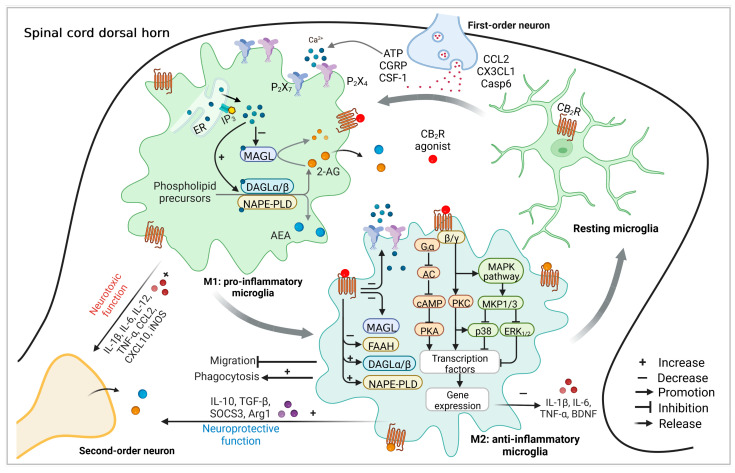
The expression profiles and possible molecular mechanisms of CB_2_R-related functional endocannabinoid system in homeostatsis and activated microglia in pain processing. When the primary afferent nerve is injured or in a state of chronic pain, the resting microglia will be activated by the mediator released from the central terminal of the primary afferent and transform into pro-inflammatory (M1) microglia. When ATP activates the increased expression of P_2_X_4_ and P_2_X_7_ on microglia, Ca^2+^ enters microglia and regulates the activities of MAGL, DAGL, and NAPE-PLD, which lead to increased production and relation of endocannabinoids such as AEA and 2-AG and pro-inflammatory mediators including IL-1β, IL-6, IL-12, IFN-γ, and TNF-α in reactive microglia. This transition was also accompanied by a distinct morphological change in the microglia, from a small soma with long, branched processes to a more amoeba-like shape. At the same time, endocannabinoid such as 2-AG or AEA and exogenous cannabinoids such as AM1241 can act on the increased expression of CB_2_R on microglia. Activation of CB_2_R can inhibit adenylate cyclase (AC), which results in a reduction of intracellular cAMP levels. Diminished cAMP level intracellularly suppresses the activity of PKA and changes the expression of respective ion channels such as P_2_X_4_ and P_2_X_7_ on microglia, leading to decreased cytosolic Ca^2+^ concentration. Changes in Ca^2+^ distribution upon CB_2_R stimulation can also regulate the activities and expressions of MAGL, DAGL, FAAH, and NAPE-PLD. Meanwhile, CB_2_R activation is also accompanied by downstream PLC activation through secondary messengers to regulate the activity of the members of the MAPK family, such as ERK_1/2_ and p38. As a final consequence, these processes can down-regulate the release of pro-inflammatory cytokines and up-regulate the release of anti-inflammatory cytokines such as IL-4, IL-10, and TGF-β by regulating the activity of different transcription factors, leading to a switch of microglia to an anti-inflammatory phenotype (M2).

In summary, the overall consequence of CB_2_R activation on microglia by endocannabinoids AEA and 2-AG or by exogenous cannabinoids appears to be to exert beneficial properties of microglia, such as the release of anti-inflammatory mediators, by promoting the generation of neuroprotective microglia phenotype (M2). The M2 phenotype could reduce neuronal hyperexcitation causally involved in central sensitization, with the capacity of phagocytosis and reduction of releasing detrimental factors like pro-inflammatory cytokines and free radicals [15,124,127,135,136,137,138,139,140,141]. The expression profiles of CB_2_R and endocannabinoids in homeostatic and activated microglia are summarized in Figure 2.

## 4. The Role of Microglial CB_2_R in Pathological Pain

### 4.1. Antinociceptive Effects of Well-Characterized CB_2_R Selective Agonists

Currently, intense interest has been focused on the use of cannabinoid compounds typically acting upon the CB_1_R and CB_2_R for the treatment of pathological pain. These widely researched compounds include endocannabinoids like AEA and 2-AG, phytocannabinoids like Δ-9-tetrahydrocannabinol and cannabidiol, as well as a large number of synthetic cannabinoids [20,142,143,144,145,146]. However, in parallel with a deeper understanding of the endocannabinoid system’s underlying expression profiles and the physiological and pharmacological properties of cannabinoid receptors, CB_2_R selective agonist compounds are increasingly recognized as safer novel therapeutic candidates, with their properties to bypass certain centrally mediated unwanted effects associated with the activation of CB_1_R. 

The following section summarizes the antinociceptive effects of various well-characterized CB_2_R selective agonists, including HU308, JWH-015, JWH-133, GW405833, AM1241, and MDA7, in different pain states to provide direct support for the hypothesis that CB_2_R can serve as a promising therapeutic target for pain relief (Table 1).

The HU308 is the first CB_2_R selective synthetic compound (K_i_ = 22.7 ± 3.9 nM) that exhibits low affinity for CB_1_R (K_i_ > 10 μM), which exerted anti-inflammatory and peripheral antinociceptive activities in an arachidonic acid-induced mouse inflammatory pain model and the late phase of the mouse formalin pain model. These activities were significantly inhibited by the use of selective CB_2_R antagonist SR144528 [147]. In the rat postoperative pain model, surgical incision-induced tactile allodynia was significantly suppressed by HU308 [148]. The topical HU308 also has been found to reduce corneal hyperalgesia and inflammation in wild-type mice, but not in CB_2_R^−/−^ mice, further validating that CB_2_R is a drug target of this compound [149].

The JWH-015, one of the earliest discovered compounds, was found to have improved selectivity for the CB_2_R (K_i_ = 13.8 nM at CB_2_R and K_i_ = 383 nM at CB_1_R) from the aminoalkylindole classification of CB_2_R agonists, which is effective in alleviating pain-related behaviors and reducing inflammatory responses without inducing psychotropic effects even when intrathecally applied [21,52,150,151,152]. The intrathecal administration of JWH015 reduced the paw incision and caused postoperative hypersensitivity and microglial activation in the spinal cord without inducing behavioral side effects. This effect was prevented by intrathecal injection of the CB_2_R selective antagonist AM630 [10,153], indicating a CB_2_R-dependent mechanism of action. In spinal nerve ligation (SNL) or lumbar 5 nerve transection (L5NT) neuropathic pain models, intrathecal JWH015 treatment significantly reduced nerve injury-induced hypersensitivity, which can also be blocked by intrathecal AM630 [13,17]. Additionally, it has also been reported that intrathecal or intraperitoneal injection of JWH-015 displayed an analgesic effect to attenuate bone cancer-induced spontaneous pain and mechanical allodynia [15,92,154].

The JWH-133 is also a well-characterized CB_2_R agonist, one of the most highly selective ligands for the CB_2_R (K_i_ = 677 ± 132 nM at CB_1_R and K_i_ = 3.4 ± 1.0 nM at CB_2_R) [155,156], which inhibits both inflammatory and neuropathic hyperalgesia through a CB_2_R-selective mechanism. For example, spinal (i.t.) or local (i.pl.) administration of JWH-133 reduced noxious mechanical stimulation evoked responses in wide dynamic range neurons recorded in SNL neuropathic pain, carrageenan-induced inflammatory pain and osteoarthritis pain, in a manner that was prevented by SR144528 [157,158,159]. The JWH-133, administered systemically (s.c.), can increase weight bearing and decrease peripheral edema or allodynia in the carrageenan-inflamed paw and osteoarthritis pain [160]. However, the JWH-133 has been shown to functionally interact with opioids to modulate antinociception in the formalin test without inducing tolerance, and can also attenuate cross-tolerance with morphine [161]. Furthermore, recent studies using CB_2_R constitutive knockout and tissue-specific genetic deletion mice suggested that self-administration of JWH-133 not only attenuated spontaneous pain and anxiety-associated behavior in the partial sciatic nerve ligation (PSNL) induced neuropathic pain model but also void of reinforcing effects in animals without pain, indicating the absence of abuse liability [14]. 

The GW405833, another highly selective CB_2_R ligand, is also classified as an aminoalkylindole [162]. In addition, the GW405833 was determined to be a selective human CB_2_R agonist in a recombinant binding assay (K_i_ = 2043 ± 183 nM at CB_1_R and K_i_ = 14 ± 6 nM at CB_2_R), while its selectivity appeared to be lower for the rat CB_2_R (K_i_ = 273 ± 42.6 nM at CB_1_R and K_i_ = 3.6 ± 1.1 nM at CB_2_R) [163,164]. In both rats and mice, pharmacological characterization of GW405833 has been previously shown to elicit efficacious antihyperalgesic and anti-inflammatory effects in several pain models, including PSNL, hind paw incision, and complete Freund’s adjuvant (CFA)-induced inflammatory pain, without eliciting the centrally CB_1_R-mediated side effects [163,165]. Similarly, systemic administration of GW405833 reduced the late phase of formalin pain and allodynia elicited by SNL in a dose-dependent manner [9]. Additionally, hind paw incision, chronic constriction injury (CCI)-induced tactile allodynia, and carrageenan-evoked peripheral edema or weight bearing were relieved by GW405833 [148,166,167]. These above-mentioned effects were demonstrated to be dependent upon CB_2_R activation rather than the activation of CB_1_R or opioid receptors by the experiments performed in CB_2_R^−/−^ mice or utilizing CB_2_R and opioid receptor selective antagonists. Interestingly, analgesic effects of high-dose GW405833 (i.p. 100 mg/kg) were also evident in the tail flick and hot plate tests in CB_2_R^−/−^ mice, which might be attributed to both moderate affinities for CB_1_R and significant CNS penetration [163,165]. Moreover, in stark contrast to treatment with WIN55,212-22, a mixed CB_1_R/CB_2_R agonist, chronic repeated injection of GW405833 was able to provide sustained reversal of allodynia following SNL without tolerance development [168]. 

The AM1241, another agonist possessing a high affinity for the CB_2_R (K_i_ = 3.4 ± 0.5 nM at the CB_2_R and K_i_ = 280 ± 41 nM at CB_1_R), belong to the aminoalkylindole class [169]. There is growing evidence supporting the hypothesis that AM1241 produced antinociceptive effects in preclinical inflammatory and neuropathic pain models lacking CNS side effects in a tetrad of behavioral tests that is used to assess cardinal signs of central CB_1_R [169]. The systemic (i.p.) and local (i.pl.) administration of AM1241 exhibited a thermal antinociceptive effect in the acute pain model of rats, which was significantly blocked by the CB_2_R selective antagonist AM630 [19]. These actions of CB_2_R have been confirmed by further studies, in which acute nociception of AM1241 was lost in the tail flick and hot plate tests of CB_2_R^−/−^ mice [22]. In the rat postoperative pain model, AM1241 also obviously suppressed tactile allodynia [148]. Moreover, systemic (i.p.) or local (i.pl.) administration of AM1241 suppressed allodynia, hyperalgesia, and peripheral edema in the carrageenan-evoked rat inflammatory pain model in a CB_2_R-dependent manner because SR144528 or AM630 specifically blocked these effects [170,171,172,173]. Similarly, intravenously administered AM1241 reduced the late phase of formalin pain, which also depends upon CB_2_R activation [9]. In the SNL or CCI of sciatic nerve-induced neuropathic pain models and the CFA-induced chronic inflammatory pain model, AM1241 (i.p., i.DRG., or i.t.) produced a significant reversal of established mechanical and thermal hypersensitivity in rats or CB_1_R^−/−^ mice [6,9,169,174]. The AM1241 could also reduce pain symptoms in a CB_2_R dependent manner in the vincristine-induced neuropathic pain model and bone the cancer-induced pain model [93,175,176].

The MDA7, one of the acylhydrazone derivatives, is a more promising CB_2_R selective agonist (*h*CB_1_R K_i_ > 10,000 nM; *h*CB_2_R K_i_ = 422 nM; *r*CB_1_R K_i_ = 2565 nM; *r*CB_2_R K_i_ = 238 nM) for the treatment of pain [24,177]. It has been shown that systemic administration of MDA7 exhibits an attenuated SNL-induced tactile allodynia in rats in a dose-dependent way. The target specificity of MDA7 was confirmed by pretreatment with selective antagonists, while attenuation of the antiallodynic effects was mediated by AM630 but not either the CB_1_R selective antagonist AM251 or the opioid antagonist naloxone [24]. This molecule has also been shown to effectively suppress mechanical allodynia rats and mice in paclitaxel (PTX)-induced neuropathic pain models. In addition, MDA7 can produce a modest thermal antinociceptive effect in naive rats without affecting locomotor activity. These effects were blocked after pretreatment with AM630 in wild type mice or were absent in CB_2_R^−/−^ mice, which indicates that the action of MDA7 directly involves the activation of CB_2_R [24,25,26]. 

As a result of the great potential of targeting CB_2_R, new potential drugs are constantly being developed. Some of them are being tested in clinical trials. For example, Olorinab, an oral and highly selective full agonist of CB_2_R, reached phase II trials for abdominal pain in Crohn’s disease and for irritable bowel syndrome [178]. However, there is still no CB_2_R selective agonist on the market as a new analgesic drug. This situation resulted from many reasons. Firstly, most CB_2_R ligands were highly lipophilic and, as such, not optimal for clinical application due to unfavorable physicochemical properties, which potentially contributed to modest or lack of clinical efficacy. Secondly, these compounds will be required to have high affinity and selectivity for CB_2_R to avoid the adverse effects of activating CB_1_R, while the target engagement of current CB_2_R ligands is poor. Aside from the development and optimization of CB_2_R ligands, the fact that human and rodent CB_2_R sequences have relatively low homology should be considered, which may give rise to differences in ligand engagement and efficacy [179]. Furthermore, preclinical pain models in animals might not fully and accurately reflect human pathological mechanisms, which may also affect the clinical translation of CB_2_R agonists. Nonetheless, substantial efforts to better optimize CB_2_R ligands for clinical application are ongoing, and many existing ligands have reached the most advanced phases, such as JBT-101 [180,181]. There is no doubt that specifically activating CB_2_R is considered a good strategy for developing new analgesic agents with fewer side effects.

**Table 1:** Efficacy of some well-characterized CB_2_R selective ligands in different pain models.

Agonist	In vitro binding profile		Pain Model	Route of Administration	Species	Efficacy	Reference
	CB_1_R	CB_2_R					
**HU308**	Ki > 10 μM Rat brain	Ki = 22.7 ± 3.9 nM Transfected cells	Formalin test	i.p., 50 mg/kg	Mouse	Antinociception	[148]
			Arachidonic acid-induced ear Inflammation	i.p., 50 mg/kg	Mouse	Reduce ear swelling	[148]
			Post-operative pain	i.p., 0.3–30 mg/kg	Rat	Antiallodynic effect	[149]
**JWH-015**	Ki = 383 nM	Ki = 13.8 nM	Tail flick test	i.p., 1–100 mg/kg	Mouse	Antinociception	[21]
			Tail immersion test/Paw pressure test	i.p., 5–20 mg/kg	Rat	Antinociception	[153]
			Formalin test	i.p., 0.1–100 mg/kg	Mouse	Antinociception	[153]
			Post-operative pain	i.t., 2–10 μg	Rat	Antiallodynic effect	[10,21,154]
				i.p., 1–10 mg/kg	Rat	Antinociception	
			Inflammatory pain (CFA)	i.p., 5–10 mg/kg	Rat	Antinociception	[153]
			Neuropathic pain (SNI, SNL, L5NT, or bone cancer induced)	i.p., 1–10 mg/kg	Rat	Antinociception	[13,15,17,21,92,155]
				i.t., 0.4–50 μg	Rat	Antiallodynic effect	
**JWH-133**	Ki = 677 ± 132 nM Rat brain	Ki = 3.4 ± 1.0 nM Human embryonic kidney 293 cells	Formalin test	i.p., 0.1–10 mg/kg	Mouse	Antinociception	[162]
			Inflammatory pain (Carrageenan or osteoarthritis induced)	s.c. 10 mg/kg	Rat	Increase weight bearing	[158,161]
				i.pl., 5–15 μg	Rat	Inhibits mechanically evoked neuron responses	
			Neuropathic pain (SNL or PSNL induced)	i.pl., 5–15 μg	Rat	Inhibits mechanically evoked neuron responses	[14,158,159,160]
				i.t., 8–486 ng	Rat	Inhibits mechanically evoked neuron responses	
				s.c. 1 mg/kg	Rat	Antiallodynic effect	
				i.v., 0.15–0.3 mg/kg	Mouse	Antinociception	
**GW405833**	Ki = 2043 ± 183 nM Cos-7 cells Ki = 273 ± 42.6 nM Rat brain	Ki = 14 ± 6 nM Cos-M6 cells Ki = 3.6 ± 1.1 nM Rat spleen	Hot plate test/Tail flick test	i.p., 100 mg/kg	Mouse	Antinociception	[166]
			Formalin test	i.v., 3–10 mg/kg	Mouse	Antinociception	[9]
			Post-operative pain	i.p., 0.3–30 mg/kg	Rat	Antiallodynic effect	[149,164]
			Inflammatory pain (Carrageenan or CFA)	i.p., 3–30 mg/kg	Mouse	Antiallodynic effect	[164,166,167]
				i.p., 0.1–100 mg/kg	Rat	Antinociception	
			Neuropathic pain model (PSNL, L5NT, or CCI)	i.p., 3–30 mg/kg	Mouse	Antiallodynic effect	[9,164,166,168,169]
				i.p., 0.01–30 mg/kg	Rat	Antiallodynic effect	
**AM1241**	Ki = 280 ± 41 nM Rat brain	Ki = 3.4 ± 0.5 nM Mouse spleen	Hargreaves acute thermal stimulation	i.p., 0.3–3 mg/kg	Mouse	Antinociception	[22]
				i.p., 0.033–0.33 mg/kg	Rat	Antinociception	[19]
			Hot plate test/Tail flick test	i.p., 0.3–10 mg/kg	Mouse	Antinociception	[22]
			Formalin test	i.p., 3–10 mg/kg	Mouse	Antinociception	[9]
				i.v., 0.3–3 mg/kg	Mouse	Antinociception	
			Post-operative pain	i.p., 3–30 mg/kg	Rat	Antiallodynic effect	[149]
			Inflammatory pain (Carrageenan, capsaicin, or CFA induced)	i.p., 0.033–1 mg/kg	Rat	Antinociception	[6,171,172,173]
				i.pl., 0.033–4mg/kg	Rat	Antinociception/Reduce paw edema	
				i.DRG, 100 nmol	Rat	Antinociception	
				i.t., 100 nmol	Rat	Antinociception	
			Neuropathic pain (SNL, bone cancer, vincristine-induced )	i.t., 0.03–0.3 μg	Mouse	Antinociception	[93,176,177] [6,9,170,175]
				i.p., 0.3–10 mg/kg	Mouse	Antiallodynic effect	
				i.p., 0.1–3 mg/kg	Rat	Antinociception	
				i.DRG, 100 nmol	Rat	Antinociception	
				i.t., 0.01–10 μg	Rat	Antinociception	
				i.v., 3–6 mg/kg	Rat	Antiallodynic effect	
**MDA7**	*h*Ki > 10,000 nM CHO-K1 cells *r*Ki = 2565 nM CHO-K1 cells	*h*Ki = 422 nM CHO-K1 cells *r*Ki = 238 nM CHO-K1 cells	Hargreaves acute thermal stimulation	i.p., 1–10 mg/kg	Rat	Antinociception	[24]
			Neuropathic pain (SNL or PTX)	i.p., 5–15 mg/kg	Rat	Antiallodynic effect	[24,25,26]

Abbreviations: CFA: complete Freund’s adjuvant; CCI: chronic constriction injury; i.t.: intratecal; i.p.: intraperitoneal; i.pl.: intraplantar; i.v.: intravenous; i.DRG.: intra Dorsal Root Ganglion, s.c.: subcutaneous; PSNL: partial sciatic nerve ligation; SNL: spinal nerve ligation; L5NT: L5 nerve transection.

### 4.2. Molecular Mechanisms Involved in the Action of Microglial CB_2_R in Pain Processing

The dorsal horn of the spinal cord is the vital site for controlling pain intensity because it is where efficient transmission of nociceptive information occurs between the central terminals of primary afferents and second-order interneurons. Additionally, the crosstalk between spinal neurons, astrocytes, oligodendrocytes, and microglia is indispensable to mediating central pain sensitization of neuronal circuits. A considerable amount of evidence has implicated the crucial role of selective agonists of CB_2_R in treating pathologic pain symptoms through the modulation of microglia in a CB_2_R dependent manner [17,25,26,29,177,182,183,184,185]. Therefore, microglial CB_2_R can serve as a promising therapeutic target for pain relief because of the important role of spinal microglia in regulating central sensitization. Later in the remainder of this review, we will focus on the molecular mechanism of targeting spinal cord CB_2_R to inhibit neuroinflammatory signaling pathways for pain relief with a microglial-centric view.

As described above, reactive microglia express CB_2_R [17,26,184]. The protein and mRNA levels of spinal cord CB_2_R were both significantly upregulated in chronic pain conditions, including CFA-induced inflammatory pain [106], SNI, SNL, CCI, and chemotherapy-induced neuropathic pain [29,30]. The above studies indicate that CB_2_R over-expression in activated microglia at the dorsal horn of the spinal cord under pathological pain conditions may occur as a result of specific neuroinflammatory responses. After that, modulation targeting CB_2_R may result in a neuroprotective effect [186]. Accordingly, the preponderance of evidence has implicated that activation of the CB_2_R system via spinal administration of CB_2_R agonists produces significant control over inflammatory and neuropathic pain in multiple models. For example, intrathecal administration of AM1241 attenuates allodynia or thermal hyperalgesia induced by CFA, SNL [6], CCI, or bone cancer models [93]. The therapeutic utility of other CB_2_R selective agonists that exhibit analgesic effects to treat different pains has been thoroughly described in Section 4.1.

Below, we will summarize our current understanding of the cellular and molecular mechanisms involved in the action of microglial CB_2_R in pain processing. However, several studies have demonstrated that spinal CB_2_R activation limits microglia activity to the ipsilateral dorsal horn, because constitutive knockout of CB_2_R results in a spread of microgliosis to the contralateral dorsal horn in an arthritis model or after sciatic nerve injury [187,188,189]. The possible mechanisms involved in this process are discussed in detail. Firstly, the activation of CB_2_R can inhibit the activities of adenylyl cyclase [190] and MAPK [174,191]. The CB_2_R selective agonists exerted anti-allodynic effects in rats by reducing MAPK (p38 and ERK_1/2_) phosphorylation and inducing MAPK phosphatases (MKP-1 and MKP-3, the major regulators of MAPKs) expression in the spinal dorsal horn [13]. The downregulation of the p38 MAPK pathway can lead to a reduction of the cytokines IL-1β, TNF-α, and brain-derived neurotrophic factor (BDNF) [174,192,193], and the suppression of ERK_1/2_ can decrease microglia proliferation [26,194,195]. Furthermore, the AMPK pathway is upregulated after CB_2_R activation, which can downregulate the synthesis of nitric oxide (NO) [196]. Actually, the activation of CB_2_R in microglial cells has been found to lead to spinal decreased iNOS, IL-6, BDNF, CCR2, and TNFα receptor expression during neuropathic pain [25,197], and increased release of anti-inflammatory cytokines, such as IL-10 [174,198,199]. Specifically, the activation of spinal CB_2_R by exercise-induced AEA release also reduces the production of IL-1β and TNFα in mice within a carrageenan-induced pain model [136]. This may also be closely related to the reduction of endocannabinoid degradative enzymes at the spinal cord level. It has been shown that intrathecal AM1241 not only modulates critical glial factors but also reduces the expression levels of MAGL, while not altering FAAH [174]. In addition, CB_2_R agonist treatment can reduce microglial purinergic receptor P_2_X_4_ upregulation [25], which may be another mechanism by which CB_2_R activation reduces microglial contributions to pain. The P_2_X_4_ was identified as a microglia-specific molecule that was activated and upregulated after peripheral nerve injury and also plays critical roles in processing nociceptive information and contributing to microglial-dependent central pain sensitization [200].

In addition to modulating microglial immune function by reducing the production of pro-inflammatory cytokines and increasing the release of anti-inflammatory cytokines involved in neuroinflammatory signaling pathways, activation of CB_2_R also can switch microglia into a more anti-inflammatory state by limiting migration and promoting phagocytic function. For example, it has been found that 2-AG can induce the recruitment of microglia partly by stimulating CB_2_R in BV-2 cells [85]. However, another study showed that CB_2_R activation in microglia stimulates MKP phosphatases, which can inhibit the ERK pathway, thus decreasing microglial chemotaxis/migration mediated by ADP [201]. Moreover, it has been found that the activation of CB_2_R is capable of inducing the removal of native beta-amyloid both in situ and in vitro by promoting the phagocytic function of macrophages [138]. On the contrary, some results also show that the activation of CB_2_R can inhibit the phagocytosis of microglia by activating ERK_1/2_/AKT-Nurr1 signal pathways [202]. These different results of microglial migration or phagocytosis mediated by CB_2_R activation may be due to the different stages of inflammation development. However, as described in Section 3 of this review, the CB_2_R activation can promote the shift of M1 to M2 microglia. All of this may enhance the beneficial properties of microglia and be associate with the restoration of microglial activity.

Overall, accumulating evidence reveals that activation of CB_2_R vitally regulates microglial immune function by blocking the normal inflammatory response with increased production of anti-inflammatory mediators and decreased production of proinflammatory mediators causally involved in central sensitization [29,31,127,135,203,204].

## 5. Conclusions

In this review article, we summarize the analgesic effects mediated by CB_2_R and the mechanisms involved in pain regulation. Firstly, it is well known that the endocannabinoid system exerts an important role in neuronal regulation. Within the CNS, CB_2_R mainly expresses in homeostatic microglia, while there is a unique feature that their expression is rapidly upregulated in activated microglia under certain pathological conditions. The CB_2_R might serve as an intriguing target for the development of drugs for the management of pain because of its ability to mediate analgesia with few psychoactive effects. Indeed, accumulating data have demonstrated that the CB_2_R agonists exert analgesic effects in various preclinical pain models, such as inflammatory and neuropathic pain. Additionally, spinal microglia can modulate the activity of spinal cord neurons and have a critical role in the development and maintenance of chronic pain. The activation of CB_2_R can reduce pain signaling by regulating the activity of spinal microglia and inhibiting neuroinflammation. Specifically, the CB_2_R activation has been reported to transform microglia from the pro-inflammatory M1 to the neuroprotective M2 phenotype by promoting the beneficial properties of microglia, such as the releasing of anti-inflammatory mediators, or the induction of phagocytosis, and reducing their ability to release pro-inflammatory cytokines involved in central sensitization. Overall, we provided an improved understanding of the underlying mechanisms involved in the action of microglial CB_2_R in pain processing. However, further studies are needed to dissect the specific role of CB_2_R expressed in different phenotype microglia to provide a better alternative to controlling pain by regulating CB_2_R.

## Data Availability

No new data were created or analyzed in this study. Data sharing is not applicable to this manuscript.

## Abbreviations

AC	Adenylyl cyclase
AEA	Arachidonoyl ethanolamide
ABDH6/12	α/β domain hydrolases 6/12
Arg-1	Arginase 1
ATP	Adenosine triphosphate
BDNF	Brain-derived neurotrophic factor
cAMP	cyclic Adenosine monophosphate
CB_1_R	Cannabinoid receptors 1
CB_2_R	Cannabinoid receptors 2
CCI	Chronic constriction injury
CFA	Complete Freund’s adjuvant
CGRP	Calcitonin gene-related peptide
CNS	Central nervous system
COX-2	Cyclooxygenase-2
CSF-1	Colony-stimulating factor-1
DAGLα/β	Diacylglycerol lipases
DRG	Dorsal root ganglion
ECS	Endocannabinoid system
FAAH	Fatty acid amino hydrolase
IFN-γ	Interferon γ
IL-1β	Interleukin 1β
IL-10	Interleukin 10
LPI	Lysophosphatidylinositol
L5NT	Lumbar 5 nerve transection
MAPK	Mitogen-activated protein kinases
NO	Nitric oxide
NAPE	N-acyl phosphatidyl ethanolamine
NAPE-PLD	N-acyl-phosphatidyl ethanolamine-phospholipase D
NAT	N-acyltransferase
PC	Phosphatidylcholine
PE	Phosphatidylethanolamine
PGE_2_	Prostaglandin E_2_
PIP_2_	Phosphatidyl inositol bis-phosphate
PLC	Phospholipase C
PNS	peripheral nervous systems
PSNL	Partial sciatic nerve ligation
PTX	Paclitaxel
SNL	Spinal nerve ligation
SOCS_3_	Suppressor of cytokine signaling 3
TGF-β	Transforming growth factor
TNF-α	Tumor necrosis factor α
2-AG	2-arachidonoyl-glycerol
